# Challenges to continuity of care among patients with long COVID-related taste and smell disorders: a qualitative study

**DOI:** 10.3389/fpubh.2026.1784507

**Published:** 2026-05-11

**Authors:** María Belén Martín-Sanz, Paloma Moro-López-Menchero, César Fernández-De-Las-Peñas, Stella Maris Gómez-Sánchez, Antonio Gil-Crujera, Laura Ceballos-García, Nuria I. Escribano-Mediavilla, Mª Victoria Fuentes-Fuentes, Lidiane Lima Florencio, Domingo Palacios-Ceña

**Affiliations:** 1 Department of Medical Specialties and Public Health, Research Group of Humanities and Qualitative Research in Health Sciences, Universidad Rey Juan Carlos, Alcorcón, Spain; 2 Department of Physical Therapy, Occupational Therapy, Physical Medicine and Rehabilitation, Research Group of Humanities and Qualitative Research in Health Science at Rey Juan Carlos University, Rey Juan Carlos University, Alcorcón, Spain; 3 Department of Physical Therapy, Occupational Therapy, Physical Medicine and Rehabilitation, Research Group of Manual Therapy, Dry Needling and Therapeutic Exercise, Universidad Rey Juan Carlos, Alcorcón, Spain; 4 Research Group GAMDES, Department of Basic Health Sciences, Universidad Rey Juan Carlos, Alcorcón, Spain; 5 Department of Nursing and Dentistry, IDIBO Research Group, Rey Juan Carlos University, Alcorcón, Spain

**Keywords:** continuity of care, long COVID, olfactory disorders, patient experience, qualitative research, taste disorders

## Abstract

Taste and smell disorders are predominant symptoms of acute SARS-CoV-2 infection and can persist in individuals with post-acute sequelae of SARS-CoV-2 infection (PASC), commonly referred to as Long COVID. These sensory impairments continue to significantly affect daily life and wellbeing, yet clinical understanding and care strategies remain insufficient. There is an ongoing need to address the specific healthcare requirements of this population. The aim of this study was to describe the experiences and perceptions of individuals with Long COVID related taste and smell disorders regarding symptom management and the challenges affecting continuity of care. A qualitative descriptive study was conducted in 2024 in the Community of Madrid (Spain) using purposive sampling. Data were collected through in-depth interviews and analyzed using thematic analysis, an approach appropriate for examining patient experiences in healthcare. Twelve participants with previously confirmed SARS-CoV-2 infection and persistent loss and/or impairment of taste and smell were interviewed. Four themes were identified: (a) Implications of Long COVID taste and smell disorders, (b) Expectations regarding prognosis and symptom cure, (c) Professional approach and symptom management, (d) Barriers and facilitators to continuity of care. Findings highlight the clinical and social relevance of taste and smell disorders in Long COVID and illustrate how qualitative methods can capture patient perspectives that may inform future mixed-methods research.

## 1 Introduction

Long COVID (LC) refers to symptoms 3 months after SARS-CoV-2 infection, lasting at least 2 months and which cannot be explained by an alternative diagnosis (1). Several hypotheses have been proposed regarding its etiology, including persistent organic damage, chronic inflammation, immune dysfunction, microthrombosis, and viral reactivation (2). This condition can affect multiple organ systems, including the lungs, heart, nervous system, and immune system, potentially leading to prolonged functional disability (1, 2). Currently, the incidence attributable to LC is estimated to be between 5 and 6% among adults who have been infected with SARS-CoV-2 (3). Although the incidence of acute COVID-19 cases has declined globally, LC and its sequelae remain clinically relevant, as persistent symptoms continue to challenge healthcare systems.

Persistent olfactory and gustatory dysfunctions, such as anosmia, hyposmia, parosmia, dysgeusia, hypogeusia, and ageusia, are among the most frequent clinical manifestations of LC, with a significant impact on patients’ quality of life (4–6). The prevalence and severity of these alterations vary depending on multiple factors, including the SARS-CoV-2 variant, the presence of pre-existing comorbidities, and the severity of the acute illness (7–9). It is estimated that the global prevalence of olfactory and gustatory dysfunction among individuals with a history of SARS-CoV-2 infection is approximately 49%, with three-year recovery rates of only 5% for olfactory function and 92% for gustatory function (10, 11). Twenty-four percent of patients who lost their sense of smell and taste due to COVID-19 did not experience full recovery (12). Although these manifestations represent a subgroup within LC, they remain susceptible to underrecognition. Their persistence continues to have disproportionate repercussions for those affected, as taste and smell play a fundamental role in human interaction with the environment and in social relationships (13). Taste and smell disorders in the context of LC have a negative psychological impact, affecting individuals’ sense of identity, interfering with daily activities (14), and leading to feelings of isolation and reduced self-esteem (15, 16). Reported consequences also include changes in food consumption, purchasing, and preparation patterns (17), loss of job opportunities, and the abandonment of occupations related to these senses (14), thereby imposing an economic burden on the household (18). Altogether, these factors result in a substantial disease burden for those affected, highlighting the critical importance of ensuring continuity of care to adequately address their clinical and care-related needs (2, 19). Nevertheless, recent studies indicate that previous research has not included long-term follow-up of patients, which limits the current clinical understanding and awareness of their care needs (19). Despite the growing body of evidence on the long-term persistence and impact of chemosensory dysfunction following SARS-CoV-2 infection, taste and smell disorders remain substantially under-recognized in clinical practice and public health planning, contributing to gaps in care and unmet patient needs (19, 20). Furthermore, earlier studies concerning healthcare provision emphasize the risk of underestimating the impact of taste and smell disorders associated with LC within the continuum of care, as these senses may be considered non-essential (17, 20). Prior research also shows that patients often feel abandoned or neglected by the healthcare system (17, 20). Given this context, it is necessary to reassess the ongoing care needs of this subgroup in order to deliver comprehensive, patient-centered care that meets their expectations and requirements (14, 20). In addition, the application of qualitative methodologies provides an essential means of capturing patients’ lived experiences and identifying barriers to continuity of care.

The aim of this study was to explore the perspectives of a group of individuals experiencing taste and smell disorders due to LC, focusing on their current illness experience and the healthcare they received, as well as the management of their symptoms by healthcare professionals.

## 2 Materials and methods

This study is part of a larger qualitative research project on taste and smell disorders in LC and continues a previously published study (21), sharing its design and methodological approach.

### 2.1 Study design

A qualitative descriptive study was conducted based on an interpretative paradigm (22), following the Standards for Reporting Qualitative Research (SRQR) (23) and the Consolidated Criteria for Reporting Qualitative Research (COREQ) (24). This study followed the principles of the Declaration of Helsinki and was approved by the ethical committee of the University Rey Juan Carlos (Code: 0907202015920).

### 2.2 Research team

Ten investigators (seven women) participated, including six physiotherapists (MBMS, PMLM, CFP, SMGS, AGC, LLF), three dentists (LCG, NEM, VFF), and one research nurse (DPC). All investigators had experience in health sciences research. None had a clinical relationship with the participants, and they did not know each other previously. Prior to the study, the positioning of the researchers was established through two briefing sessions that addressed the theoretical framework of this qualitative study, their beliefs and their motivation for the research. Given the researchers’ professional backgrounds in health sciences, their prior knowledge and disciplinary perspectives may have influenced data interpretation. To address this, reflexive discussions were maintained throughout the research process in order to critically examine assumptions, contrast interpretations, and enhance analytical transparency (25). Researcher reflexivity was encouraged through reflexive reports, which helped the researchers monitor their ideas, biases, and reflections throughout the research process (26).

### 2.3 Participants, context, and sampling strategies

A non-probabilistic purposive sampling approach was used (22, 27). Participants were recruited from two COVID-19 patient organizations (Long COVID ACTS and Persistent COVID Spain) through flyers, social media, and online platforms for COVID-19 support groups. The inclusion criteria consisted of individuals aged 18 years or older who had survived COVID-19 and were experiencing persistent smell and/or taste disorders (total or partial loss) at the time of recruitment, confirmed by a specialist physician in otolaryngology. Exclusion criteria included individuals with pre-existing neurological or sensory disorders affecting taste or smell, individuals with communication impairments that could interfere with the interview process, and individuals with acute COVID-19 infection. Long COVID was defined according to the WHO criteria as symptoms that persist or appear 3 months after the initial SARS-CoV-2 infection and last for at least 2 months without an alternative explanation (28). A total of 12 participants were included. No participants refused to participate or dropped out. All participants provided written informed consent to take part in the study. Additionally, written consent was obtained to use their narratives for the purposes of reporting the present findings, while ensuring participant anonymity.

### 2.4 Data collection

Data were collected through semi-structured interviews, based on a question guide, and researcher field notes (Table 1) (22, 27) in the Community of Madrid (Spain) during the first trimester of 2024. During the interview, researchers noted key words and themes and retrieved them through related questions to clarify content (22, 27). Interviews were conducted through the Microsoft Teams digital platform and also in person, in a private room. Twelve interviews were conducted: six online and six face-to-face. A total of 531 min of interviews were recorded and transcribed verbatim. Field notes were employed as an additional source of contextual information. The interviews were conducted by PMLM, MBMS, SMGS, and AGC.

**Table 1:** Semi-structured question guide.

Area of study	Question
Expectations of prognosis and cure	What is your perspective on having taste and smell disorders due to persistent COVID? What is most relevant to you? What do you feel are the daily implications? What are your expectations on the evolution of the disease, and on symptom management?
Professional approach	What was the professional approach to the symptoms by healthcare professionals? What was most relevant for you? In your opinion, what should healthcare professionals take into account when treating people with this problem?
Barriers and facilitators to continuity of care	How do you perceive the continuity of care of Long COVID in healthcare organizations? What barriers influence the continuity of care in taste and smell disorders? What elements facilitate it? How do you think that healthcare can be guaranteed for patients with these symptoms?

Data collection ceased when thematic saturation was reached, meaning that no new data themes emerged from the information obtained from the interviews (27, 29).

### 2.5 Analysis

The interviews were analyzed using an inductive thematic analysis (27, 30). The in-depth interviews and the researcher’s field notes were transcribed. A thematic analysis of the transcripts was carried out by identifying the text fragments that provided relevant information to answer the objectives (30, 31). From these text fragments, codes were identified by two researchers. Subsequently, the codes were grouped into categories according to the similarity of content (30, 31). From the categories, the final themes were obtained. For greater clarity, a matrix was constructed using the results obtained from the analysis (32). To identify the themes, the results were combined through joint meetings of the research team. In the event of disagreements, team discussions were held to reach a consensus. No qualitative software was applied for data analysis. However, the coding and thematic analysis were conducted systematically using Microsoft Excel (Microsoft Corp., Redmond, WA), which allowed the research team to organize transcript excerpts and group them into defined codes, categories, subthemes, and themes. Moreover, the researchers used the same Excel template shared via OneDrive (Microsoft Corp., Redmond, WA) to simultaneously modify, highlight, and extract the results of the coding process as part of a collaborative network.

### 2.6 Rigor

The trustworthiness criteria of Guba and Lincoln were applied (33). The techniques used are listed in Table 2.

**Table 2:** Trustworthiness criteria.

Criteria	Techniques performed and application procedures
Credibility	Investigator triangulation: each interview was analyzed by two researchers.
	Triangulation of data collection methods: semi-structured interviews were conducted, and researcher field notes were kept.
	Member checking: the participants were asked to confirm the data obtained. All participants were offered the opportunity to review the audio and/or video records to confirm their experience. None of the participants made additional comments.
Transferability	In-depth descriptions of the study were performed, providing details of the characteristics of researchers, participants, contexts, sampling strategies, and the data collection and analysis procedures.
Dependability	Audit by an external researcher: an external researcher assessed the study research protocol, focusing on aspects concerning the methods applied.
Confirmability	Data collection and analysis triangulation. Researcher reflexivity was encouraged via the completion of reflexive reports.

## 3 Results

Most participants were women (75%) with a mean age of 55.25 years (SD = 8.20). In addition to taste and smell disorders, common persistent symptoms included fatigue (58.4%), sleep disturbances (75%), and mental agitation, anxiety or fear (83.3%). Memory loss, muscle weakness, exertional dyspnea, and muscle pain or weakness were also reported. Less frequent symptoms (16.7%) included resting dyspnea, hair loss, palpitations, skin rashes, and mental slowness.

One-third of participants reported three additional post-COVID-19 symptoms, and 25% reported five or more. According to the Functional Impairment Checklist (FIC) (34), most participants reported no limitations in basic (83.3%) or instrumental (91.7%) daily activities. However, 41.6% experienced some degree of work-related limitation, and 50% reported restrictions in leisure activities.

### 3.1 Results of the thematic analysis

Four themes were identified: (a) Implications of LC taste and smell disorders, (b) Expectations regarding prognosis and symptom cure, (c) Professional approach and symptom management, (d) Barriers and facilitators to continuity of care (Table 3). Participant quotations are provided in the Supplementary file S1.

**Table 3:** Themes and categories.

Themes	Categories
Implications of Long COVID taste and smell disorders	Emotional consequences Social consequences Consequences for personal safety and integrity
Expectations regarding prognosis and symptom cure	
Professional approach and symptom management	Lack of clinical knowledge on evolution, prognosis and treatments Downplaying symptoms
Barriers and facilitators to continuity of care	Organizational barriers to continuity of care Delay and avoidance in seeking help Facilitators of continuity of care

### 3.2 Implications of long COVID taste and smell disorders

#### 3.2.1 Emotional consequences

Participants described emotional instability and distress. They experienced feelings of overwhelm, not knowing how to manage their symptoms while gaining awareness of the importance of these senses in their daily lives. In addition, participants recounted feelings of frustration, anger and helplessness, in the face of uncertainty and the permanence of symptoms over time.

In addition, some participants perceived their symptoms as disabling; they also reported fear and anxiety, and these emotions were intensified by a sense of abandonment by the healthcare system.

The participants felt enraged about the management and course of their condition. In addition, there was concern about the possible long-term consequences of the permanence of the symptoms, since it was a new disease, and they did not know how it would evolve over time.

#### 3.2.2 Social consequences

The presence of symptoms disrupted the social life of the participants and their relationships with friends and family. Social participation limitations were acknowledged, as many of the celebrations with family and friends are based on food, and the participants could not fully enjoy these events because of their symptoms, feeling isolated and socially disconnected.

Many participants described a reduced interest in social activities based on or related to gastronomy, redirecting their leisure toward other cultural activities (e.g., going to the theater or cinema).

#### 3.2.3 Consequences for personal safety and integrity

The loss of taste and smell was perceived by participants as a risk to their physical integrity. The participants described great concern about potential dangers that might go undetected when these senses are altered, such as fire (house fire) or gas leakage. In addition, they reported an increased sense of dependence on family members when living alone.

Another concern voiced by participants was suffering from food poisoning because of not detecting spoiled food. This led some people to modify their routines such as the choice of restaurants, where they began to rely on the reviews of other consumers to feel confident.

### 3.3 Expectations regarding prognosis and symptom cure

The participants narrated that they had low expectations of being cured or improving their prognosis, due to the time elapsed without changes or improvement in their symptoms. They reported having resigned themselves to not recovering taste and smell, due to the lack of perceived effects and changes from treatment. Many stated that they “have gotten used to the idea that recovery may never occur”. They expressed limited hope that changes can be made to improve their current status.

Over time, the participants showed a loss of confidence in their recovery. Their initial expectations were based on small changes and the initial improvements they felt for their recovery. The delay in improvement meant that many participants felt that recovery may take longer and may not even be complete. This forced them to accept that they may not be able to fully recover at all:

Many participants reported feeling resigned, understanding that they can do nothing but wait and accept their current situation, considering these sequelae permanent.

Some participants felt they have to accept this after-effect as their “dues” for having survived COVID-19 without having suffered more severe symptoms.

### 3.4 Professional approach and symptom management

#### 3.4.1 Lack of clinical knowledge about evolution, prognosis, and treatments

The participants stated that health professionals still had a great lack of knowledge regarding how to manage LC loss of taste and smell. They realized that the professionals were unaware of the medical reasons that influence the evolution and prognosis of their symptoms. The professionals were also uncertain about treatments for the improvement or rehabilitation of taste or smell.

Most participants stated that the professionals recommended them to wait it out as the only solution. However, comments such as “it will pass” failed to reassure the participants. Some professionals even expressed their surprise when they were told that they still had these symptoms. In addition, the specialists (ENT, Internal Medicine, Occupational Medicine, Neurology and Pneumology) failed to inform them or refer them for specific taste and smell rehabilitation. One participant (P. 3) reported how difficult it was to receive any type of olfactory rehabilitation, because physicians were unaware that this service was available within the health care system. This generated the need for participants to seek other options for improvement or rehabilitation outside of medical recommendations, such as information from family/friends or the Internet. This led them to the use of self-treatments, such as smelling perfumes at home, trying to decipher smells and tastes with their eyes closed, or engaging in mental visualizations.

#### 3.4.2 Downplaying symptoms

Our participants narrated how professionals did not pay enough attention to their symptoms. Professionals focused on the care of more “severe” sequelae, considering taste and smell symptoms as minor.

For one participant (P. 11), the professionals only noted her symptoms in her medical history with no attempt to give her an answer, since she was experiencing the lowest level of potential sequelae.

These experiences were in stark contrast with the feelings of the participants, who felt that their symptoms were of great relevance and impact. One participant (P. 5) angrily recounted how a physician was surprised that she was looking for a solution to these symptoms given that she was a health professional.

According to participants, one of the reasons for this low importance given by healthcare professionals is that it does not affect life, nor does it seriously limit the person’s day-to-day life, describing it as a “minor blip” in the patient’s medical history:

### 3.5 Barriers and facilitators to continuity of care

#### 3.5.1 Organizational barriers to continuity of care

Among the barriers that hinder the management of people with taste and smell symptoms due to LC within the healthcare organization, the lack of medical control and follow-up of LC symptoms was identified. The patients perceived a certain disbelief, together with lack of accompaniment and follow-up by health professionals, defining the clinical management as “embarrassing.”

Only those LC symptoms categorized as “rare,” “limiting,” or “painful” are followed up, thus their symptoms go unanswered. One participant (P. 9) stated that the reason for the lack of follow-up is the economic impact it would have on the healthcare system to respond to all patients with LC.

Another barrier to continuity of care was the overload of the healthcare system, which limited access to medical appointments in primary care and specialist services, leading some patients to abandon attempts to seek care.

A further difficulty faced by the participants was access to specialist physicians in public hospitals. This was because primary care physicians did not think it was the right time to refer them to a specialist or because such referral was not necessary.

The most important barrier, however, was the prolonged delay before participants were assessed by healthcare professionals, which sometimes led to their symptoms being overlooked in subsequent consultations.

#### 3.5.2 Delay and avoidance in seeking help

Some participants reported that they sometimes avoided or did not seek professional health care for their sequelae. The first of the reasons reported was to avoid bothering health professionals with these symptoms, as they appreciated the professional overload in the context of health care collapse after the COVID-19 pandemic. In addition, they did not seek help because they “sympathized” with people with more serious sequelae and did not want to harass health professionals.

Another reason for not seeking help was to consider that their symptoms were not serious or relevant or simply staying alive was reason enough not to seek help. Comparing themselves with other people who were infected and had worse prognosis, they were grateful for only suffering taste and smell problems, considering their symptoms as “lower priority side effects” or “less serious” problems, thereby downplaying the loss of these senses.

As a result, they felt that their symptoms were not severe and/or relevant enough to be discussed with healthcare professionals, even though the symptoms were still present and continued over time.

#### 3.5.3 Facilitators of continuity of care

The participants described certain aspects that facilitated proximity between patients and professionals, improving health care. These included sensing the professionals’ interest in their symptoms, giving importance to the sequelae and trying to provide a response to the clinical situation.

Supporting and being present with the patients, comforting them and treating them with kindness, helped recovery and made it easier for the participants to express their concerns to the professionals. Finally, participants valued positively the information conveyed by the professionals on the progress or new developments in recovery, or regarding the progression of the symptoms, or providing examples of other cases similar to those of the participants.

## 4 Discussion

Consistent with previous studies, our results highlight the emotional and social impact of LC taste and smell symptoms. Previous studies describe that the emotional impact of taste and smell symptoms maintained over time is associated with anxiety and depression (35, 36). Long COVID as a new disease increases distress and uncertainty, with feelings of guilt, helplessness and loss (37). These symptoms have a negative impact on the perceived quality of life and subjective self-perceived health (7, 38).

In line with previous literature, fear was also a prominent feeling among people with LC-related taste and smell symptoms. O’Brien et al. (39) described how fear is not only associated with the severity of the symptoms, but also with the temporary permanence of the symptoms, causing lack of hope for a possible recovery.

These findings also confirm earlier reports showing that loss and/or alteration of taste and smell generates stress, anxiety, and greater vulnerability due to reduced protection against domestic hazards such as chemicals, gas, or fire (14, 36). This leads to an increased sense of dependency on family and friends as the ability to detect potential risks is reduced (40).

Similarly, our findings support previous research showing that impairment of these senses hinders social interactions and personal relationships (6, 37). Previous studies show the negative implications that patients with taste and smell impairment due to COVID-19 suffer in their social relationships, generating feelings of social isolation (35–37). Javed et al. (36) explained that people limit attendance to social gatherings related to food. Patients become distressed as they no longer enjoy food (35, 37). These consequences can affect the comfort experienced by the person in their own social and cultural environment, impacting their own identity and social role (37).

Consistent with previous studies, the prolonged duration of symptoms led participants’ expectations of recovery and improvement to decline over time. Previous studies show the loss of hope for symptom management in patients with LC, accepting that there is little chance of recovery in the short term (14, 37).

Regarding professional management, participants perceived a lack of professional knowledge about the evolution, prognosis, or treatment of these symptoms. Only a small proportion of participants had been assessed by medical specialists. Those who had been seen reported that healthcare professionals did not provide clear answers regarding their clinical uncertainty. Previous studies describe how professionals were unable to recognize or identify whether smell and taste symptoms originated from LC (20, 37, 41) or they gave limited or contradictory clinical recommendations (8). Beyond confirming these earlier reports, our findings further highlight how this uncertainty affects patients’ sense of being clinically unsupported and contributes to difficulties in continuity of care.

Other authors (20, 21, 42) state that patients feel that professionals are baffled by this symptomatology and its fluctuating nature and are unable to help them. This causes patients to disbelieve and mistrust healthcare professionals, thus they avoid sharing their symptoms and prefer to seek help through other non-professional means, such as websites or social networks (37). Our findings extend this understanding by showing that these experiences may also contribute to delayed help-seeking and to the normalization or minimization of symptoms by patients themselves.

Our participants reported that health professionals tended to downplay the importance of their symptoms. Burges et al. (42) describe how patients with LC felt a lack of empathy, understanding and support for their symptoms on behalf of professionals. There is a risk of “invisibility” of the sequelae of LC and lack of validation, and they may become “forgotten” or “overshadowed” by other symptoms, remaining overlooked in health care (35, 42). Fang et al. (20) exposed the risk of professionals focusing on “clinically severe and visible symptoms” while forgetting the impact of these sequelae and the implications for those who suffer them. Leggat et al. (37) reported that patients with LC sequelae experienced difficulties in reporting their symptoms to healthcare professionals, describing this process as a “challenge.” These experiences generated feelings of abandonment among patients. In addition to confirming this, our findings suggest that the perceived trivialization of taste and smell disorders may reinforce the idea that these symptoms are a lower clinical priority, despite their substantial emotional and social consequences.

In addition, our results show a paradox effect. Although a group of patients claimed lack of medical attention for downplaying the importance of their symptoms, there were patients who did not seek help because they considered that their symptoms did not justify this attention compared to other patients suffering more serious sequelae. Burges et al. (42) showed this same situation in their results, stating that patients do not report these symptoms to professionals because it is difficult for them to explain them, because they feel guilty for still having these symptoms or because they feel that they are not important, while at the same time they expressed in their study “tears of joy and relief” when they saw that they were cared for and that attention was paid to these symptoms. Also, Burges et al. (42) report that their participants were labeled as “dramatic” in terms of how they experienced their symptoms. This caused them to minimize their symptoms. Burges et al. (42) describe this as “the dramatic burden of suffering from an invisible condition”. This differs from the findings by Turk et al. (43), where patients with LC symptoms described their distress when considering themselves “eligible” for care, yet not actually receiving such care.

One of the main barriers identified in the continuous management of taste and smell symptoms is the lack of continuity of care, due to the absence of an established agreement on the duration for which LC should be monitored (14, 43). In addition, communication between primary and specialized care physicians can lead to gaps in the continuity of care. Previous studies (14, 20, 37, 43) have highlighted the difficulty experienced by patients with LC sequelae to access specialized care services. These patients are unable to obtain referrals or suffer long waiting times (20, 43). O’Hare et al. (44) showed that the absence of agreed follow-up between different levels of care leads to fragmentation of care and generates unnecessary burden on patients. Burges et al. (42) described how when patients with LC taste and smell symptoms do not find their care needs resolved, there is an increase in self-treatment and remedy seeking outside of medical care, from remedies or the internet. Our findings add nuance to this literature by showing that, for participants with persistent taste and smell disorders, these organizational barriers were experienced not only as delays in access, but also as a form of clinical invisibility.

Our results coincide with those reported by Turk et al. (43) by stating that there are barriers to care for the sequelae of LC in what they call “health care access filters” (recognition of the health problem and help seeking by the patient, recognition of the problem by the primary care health professional, diagnostic management, treatment and referral, and access to specialized care).

A key aspect of meeting the needs of patients with LC is to improve coordination between primary and specialty care to improve continuity of care (20, 43). Healthcare processes that guarantee the quality of care for these patients in terms of diagnosis, evaluation, follow-up and treatment must be guaranteed (2, 44). The facilitators for this continuity of care for those with taste and smell disorders due to LC include personalized management strategies (6, 14), understanding and clinical empathy (6, 14, 44) or active listening and collaborative problem solving between professionals and patients (43).

Beyond these clinical and organizational challenges, our findings reinforce that taste and smell disorders related to LC represent a subgroup of patients who bear a disproportionate psychosocial and economic burden and remain susceptible to underrecognition. Recent evidence confirms that these dysfunctions can persist for years after infection, raising the risk of invisibility within healthcare systems (9). Qualitative approaches, such as those used in this study, are particularly suited to capturing patients’ lived experiences and to identifying barriers in continuity of care that are not easily captured by quantitative research (2). Although clinical and epidemiological conditions of the pandemic have evolved, recent studies continue to report persistent olfactory and gustatory dysfunctions in individuals with LC (3, 14). This confirms the ongoing relevance of our findings and underscores the importance of maintaining attention on this subgroup of patients. Future mixed-methods studies could integrate qualitative evidence with clinical and epidemiological data to contribute to the development of care strategies that recognize the impact of these sequelae in this subgroup of patients.

Finally, from a public health and service organization perspective, these findings highlight the need for greater recognition of persistent taste and smell disorders as part of LC care. Clearer referral pathways and better coordination between primary and specialist care may help reduce delays, uncertainty, and fragmentation in the care process. In addition, multidisciplinary approaches may be useful in addressing the functional, emotional, and healthcare needs associated with these persistent symptoms.

### 4.1 Strengths and limitations

The main strength of this study lies in its qualitative approach to a topic that remains underexplored, allowing for an in-depth understanding of patients’ experiences. The inclusion of a homogeneous sample with clearly defined criteria and a well-justified sample size further strengthen the credibility of the findings. Analytical rigor was enhanced through an iterative coding process and team-based discussions, supporting consistency in theme development. However, the study also presents certain limitations, such as the potential for selection bias and limited transferability of the findings, which is inherent to qualitative research, as the results are closely linked to the specific context and characteristics of the participants. For this reason, this study has outlined the context of LC management and care in Spain, thereby providing information that allows for the assessment and evaluation of similarities with other contexts (26, 45). In addition, participants’ experiences were shaped by the healthcare context in which the study was conducted, and differences in healthcare organization, referral pathways, and access to services should be considered when interpreting these findings in other settings. Regarding sample size, this study included 12 participants. Although such a number may be considered small from a quantitative standpoint, in qualitative research adequacy is determined by the information power of the sample rather than its absolute size (46). Moreover, methodological evidence indicates that content saturation is typically achieved with samples ranging from 9 to 17 participants (47). In our study, the sample yielded data that were sufficiently rich and aligned with the analytical aims. Another key aspect was that half of the participants completed the interviews online, while the other half participated in a face-to-face format. Those who took part in the online interviews felt comfortable and did not experience difficulties in sharing their experiences. The authors believe that offering participants the flexibility to choose the interview modality enabled individuals with LC who might have difficulty attending an interview outside their home to participate, and allowed researchers to adapt to their individual circumstances. In addition, the findings are based on participants’ narratives, which may have been influenced by retrospective interpretation and the emotional burden associated with persistent symptoms. However, these subjective accounts were central to the aim of the qualitative study, which sought to explore patients’ experiences of persistent taste and smell disorders and their implications for continuity of care (48–50). Despite these limitations, the findings offer relevant insights to inform patient-centered approaches and continuity of care in LC services.

## 5 Conclusion

The implications of LC taste and smell disorders have a significant perceived impact on emotions and the ability to relate to their social environment. Furthermore, it is necessary to ensure continuity in the care of those affected, due to the presence of barriers in health organizations and among professionals themselves to effectively address the symptoms and improve the care process.

## Data Availability

The raw data supporting the conclusions of this article will be made available by the authors, without undue reservation.
