# Supplementary material for: Challenges to continuity of care among patients with long COVID-related taste and smell disorders: a qualitative study
Source: Front Public Health. 2026 May 11;14:1784507. doi: 10.3389/fpubh.2026.1784507 (PMC13199319; doi:10.3389/fpubh.2026.1784507)
Supplement: Supplementary file 1 [file Table_1.docx]

**S1: Participant quotations**

| **Themes** | **Categories** | **Narratives** |
| --- | --- | --- |
| Implications of Long COVID taste and smell disorders | Emotional consequences | *You see that you are going to do something and it’s invalidating, because it affects you psychologically. And I have to say that nothing is wrong with me, that I’m fine, that I’m here. Well, it's all an act and I'm afraid. You feel abandoned, very abandoned. (P.11)*  *I hope it won’t have any major consequences. As we don't know anything about it, in a few years it could be worse. (P.11).* |
|  | Social consequences | *Things change. Before I was more inclined to going out to lunch, going out for dinner, but when you don't taste the flavor, you lose that kind of thing... You must change your plans. (P.12).*  *You lose the desire to eat, because although the moment may be joyful, a happy moment when you are with your family, I cannot fully enjoy it because I can't taste anything. (P.8).* |
|  | Consequences for personal safety and integrity | *I'm afraid of falling asleep, for a fire to start and not realize it. It scares me, it worries me because if something happens at home and I'm alone... A sense of smell can help save your life. (P. 12).*  *I’m very careful not to eat in a place where I am not 100% sure, because I might eat something that has gone off and I won't notice it. (P. 9).* |
| Expectations regarding prognosis and symptom cure |  | *I have recovered up to what I have recovered, and I don't know if I will recover anything else. If you recover fine, and if not, then accept it… (P. 5). It would be great to recover both smell and taste. But no, I don't have much hope. I'm in a rut. (P. 12).*  *I have already assimilated it because since it’s been a long time, I don't know if I will recover it. Obviously, I would love to have 100% smell and taste, but after such a long time I don't know if it will be possible. (P. 9).*  *I have convinced myself that I can't do any more (...) I don't beat myself by saying “Oh, look what I have left”. I have learned to live with it. That's all. (P. 3).*  *It's like the customs duty you pay for going through COVID and surviving. (P. 12).* |
| Professional approach and symptom management | Lack of clinical knowledge on evolution, prognosis and treatments | *In summary, Will this improve? The response they give me... “Well, we don't know.” (P. 11).*  *I read that there was a rehabilitation (...) I told my doctor about it, but he had no idea about it. If they don't know about it, they won't refer me. (P. 6).* |
|  | Downplaying symptoms | *Since there are more serious sequelae than this one, they do not give it priority. Because there are people who have been very ill with other symptoms, therefore this is something very minor and they don't give it importance. (P. 9).*  *They take it as a joke, an anecdote. They downplayed this symptom and always compared it to the fact that it was better than being at home with oxygen like other patients. (P. 3).* |
| Barriers and facilitators to continuity of care | Organizational barriers to continuity of care | *I don't have anything, no follow-ups with anyone, no doctors. In the end nobody listens to you. (P. 8).*  *I asked him to send me to the ENT and he told me that it was too soon, to wait. I told him that the sooner I was diagnosed, the better to start treatment, and he told me that I still had a margin of 24 months until he would refer me... (P. 5).*  *Nobody gives it the same importance because it has been forgotten. As a long time has passed, and nothing happens, well, that's it, nothing is done. But those of us who suffer from it continue to experience the symptoms daily. (P. 8).* |
|  | Delay and avoidance in seeking help | *It was out of solidarity and not to cause discomfort. My situation was nothing compared to all the consequences and everything that the Spanish healthcare system was going through with COVID. The priority was people unable to walk or talk or with more serious consequences. Obviously, what was happening to me was absolutely nothing. I’m not going to be nagging my family doctor about something when I knew the system was brimming with cases. (P. 9).*  *I survived the first wave, which was the worst, and at least I'm alive. Because all this is nothing after what was seen in the hospitals. (P. 6).* |
|  | Facilitators of continuity of care | *If they had shown interest, they would have helped me, in the end it is to improve my quality of life a little. Even if they can't do anything, feeling that you matter to someone helps you to cope a little better. (P. 8).* |
