# Supplementary material for: Challenges to continuity of care among patients with long COVID-related taste and smell disorders: a qualitative study
Source: Front Public Health. 2026 May 11;14:1784507. doi: 10.3389/fpubh.2026.1784507 (PMC13199319; doi:10.3389/fpubh.2026.1784507)
Supplement: Supplementary file 2 [file Table_2.docx]

**Additional file: Healthcare system context relevant to the continuity of care for Long COVID in Spain**

| **Contextual factor** | **Description** |
| --- | --- |
| **National healthcare system** | Spain has a universal, tax-funded National Health System (Sistema Nacional de Salud, SNS) providing healthcare coverage to all residents. Healthcare delivery is predominantly public and organised around primary care and specialised hospital care (1). |
| **Governance structure** | Healthcare governance in Spain is decentralised. The Ministry of Health defines national strategic frameworks and coordination mechanisms, while the autonomous communities are responsible for healthcare planning, organisation of services, and allocation of resources (2). |
| **Recognition of Long COVID** | Long COVID has been internationally recognised and incorporated into disease classification systems (ICD-10 code U09.9), contributing to the formal identification and clinical management of persistent symptoms following SARS-CoV-2 infection (3). |
| **National strategic framework** | Spain’s national strategy for chronic disease management promotes coordinated, person-centred and multidisciplinary care, as well as continuity of care across healthcare levels. Although published after data collection period, it provides relevant policy context for interpreting the organisation and prioritisation of care for conditions such as Long COVID (4). |
| **Organisation of Long COVID care** | In Spain, there is currently no nationally designated network of specialised Long COVID reference centres. Clinical management is generally integrated within existing hospital specialties and coordinated with primary care services, with organisational models varying across regions and hospitals (5). |
| **Role of primary care** | Within the Spanish healthcare system, primary care acts as the usual first point of contact and as a gatekeeper to specialised services. Clinical guidance for Long COVID recommends that patients with persistent symptoms are initially assessed in primary care and referred to specialised services when necessary, thereby playing a central role in ensuring continuity of care across levels (2,5). |
| **Research initiatives** | In Spain, collaborative research initiatives have contributed to advancing knowledge and organisation of Long COVID care. The Spanish Network for Research on Long COVID (REiCOP – Red Española de Investigación en COVID Persistente) promotes collaboration between researchers, clinicians and patient organisations and has highlighted the importance of structured care pathways and multidisciplinary management for individuals with persistent symptoms following SARS-CoV-2 infection (6). |
| **Patient organisations** | Patient-led organisations have also contributed to raising awareness and advocating for recognition and improved care for individuals with Long COVID. National groups such as Long COVID ACTS and COVID Persistente España have supported patient representation, visibility and participation in research initiatives related to persistent COVID-19 symptoms (7). |

Table note: This table summarises contextual factors about the organisation of the Spanish healthcare system to support interpretation of the study findings and assessment of transferability to other healthcare settings.

**References:**

1. Ley 14/1986, de 25 de abril, General de Sanidad. Boletín Oficial del Estado. 1986;102:15207–15224. https://www.boe.es/boe/dias/1986/04/29/pdfs/A15207-15224.pdf [Access 17 March 2026]
2. Ley 16/2003, de 28 de mayo, de cohesión y calidad del Sistema Nacional de Salud. Boletín Oficial del Estado. 2003;128:20567–20588. https://www.boe.es/boe/dias/2003/05/29/pdfs/A20567-20588.pdf [Access 17 March 2026]
3. World Health Organization. A clinical case definition of post COVID-19 condition by a Delphi consensus, 6 October 2021. Geneva: World Health Organization; 2021. https://www.who.int/publications/i/item/WHO-2019-nCoV-Post_COVID-19_condition-Clinical_case_definition-2021.1 [Access 17 March 2026]
4. Ministerio de Sanidad. Estrategia para el Abordaje de la Cronicidad en el Sistema Nacional de Salud. Documento de desarrollo 2025–2028. Madrid: Ministerio de Sanidad; 2025. https://www.sanidad.gob.es/areas/calidadAsistencial/estrategias/abordajeCronicidad/docs/20250704_EAC_DOCUMENTO-DESARROLLO_2025-2028_Final.pdf [Access 17 March 2026]
5. Sociedad Española de Médicos Generales y de Familia (SEMG). Guía clínica para la atención al paciente Long COVID / COVID persistente. Madrid: SEMG; 2021. https://www.semg.es/images/2021/Documentos/GUIA_CLINICA_COVID_Persistent_20210501_version_final.pdf [Access 17 March 2026]
6. Red Española de Investigación en COVID Persistente (REiCOP). Declaración de El Escorial. 2023. https://www.semg.es/images/2023/documentos/Declaracion_escorial_reicop.pdf [Access 17 March 2026]
7. Long COVID ACTS. Long COVID ACTS – Asociación de afectados por COVID persistente. Long COVID Autonomous Communities Together Spain. Dossier Informativo Long COVID / COVID Persistente. https://frecuenciaenfermera.es/wp-content/uploads/2021/07/Dossier-LONGCOVIDACTS-1.0.pdf [Access 17 March 2026]
